# High Prevalence of *bla*_CTX-M-15_ Gene among Extended-Spectrum β-Lactamase-Producing *Escherichia coli* Isolates Causing Extraintestinal Infections in Bangladesh

**DOI:** 10.3390/antibiotics9110796

**Published:** 2020-11-11

**Authors:** Razib Mazumder, Ahmed Abdullah, Dilruba Ahmed, Arif Hussain

**Affiliations:** Laboratory Sciences and Services Division, International Centre for Diarrhoeal Disease Research, Bangladesh (icddr,b), Dhaka-1212, Bangladesh; rmazumder@icddrb.org (R.M.); ahmed.abdullah@icddrb.org (A.A.); dahmed@icddrb.org (D.A.)

**Keywords:** *Escherichia coli*, Bangladesh, ST131, urinary tract infections, virulence genes, *bla*_CTX-M-15_, carbapenem resistance

## Abstract

The emergence of multidrug-resistant (MDR) *Escherichia coli* (*E. coli*) clonal lineages with high virulence potential is alarming. Lack of sufficient data on molecular epidemiology of such pathogens from countries with high infection burden, such as Bangladesh, hinders management and infection control measures. In this study, we assessed the population structure, virulence potential and antimicrobial susceptibility of clinical *E. coli* isolates from Dhaka, Bangladesh. A high prevalence of MDR (69%) and extended-spectrum β-lactamase production (ESBL) (51%) was found. Most *E. coli* isolates were susceptible to amikacin (95%), meropenem (94%) and nitrofurantoin (89%) antibiotics. A high prevalence of ST131 (22%) and ST95 (9%) followed by ST69 (4%) and ST73 (3%) was observed. Phylogroups B2 (46%), B1 (16%), D (10%) and F (9%) were prominent. *bla*_CTX-M-15_ (52%) and *bla*_NDM-1_ (5%) were the most prevalent ESBL and carbapenem resistance genes, respectively. Moreover, the predominant pathotype identified was extraintestinal pathogenic *E. coli* (ExPEC) (41%) followed by enteric pathogens (11%). In conclusion, our results suggest the transmission of clonal *E. coli* groups amidst diverse *E. coli* population that are associated with high virulence potential and MDR phenotype. This is of high concern and mandates more efforts towards molecular surveillance of antimicrobial resistance (AMR) in clinically significant pathogens.

## 1. Introduction

Urinary tract infection (UTI) is one of the most common bacterial infections, which has become a major public health burden worldwide [1]. The most frequent causative agent of community-acquired UTIs is uropathogenic *E. coli* (UPEC) accounting for 75–85% of infections [1]. As an extraintestinal pathogen, *E. coli* is also frequently implicated in bloodstream infections and neonatal meningitis [2,3]. The emergence of multidrug-resistant (MDR) *E. coli* producing extended-spectrum β-lactamase (ESBL) enzymes and carbapenemases has made empirical therapy void, leading to high morbidity and mortality [4]. Strikingly, an increasing number of ExPEC strains producing β-lactamase such as CTX-M-15 is being associated with clonal lineages such as ST131 [5]. Isolates belonging to this lineage have spread extensively, and they are responsible for the rapid worldwide increase in the prevalence of bloodstream and urinary tract infections. Moreover, the development of resistance to carbapenems among such pathogens is the greatest threat to public health because there are still no effective and safe therapeutic options available for treating carbapenem-resistant pathogens causing life-threatening infections [6].

Currently, the CTX-M-group enzymes that belong to class A ESBLs are the most predominant ESBLs worldwide, including the developing world. The genes for these ESBLs were detected on several plasmid incompatibility groups but are sometimes also present on chromosomes [7]. There are more than 50 allelic variants, which are clustered into six groups: CTX-M-1, CTX-M-2, CTX-M-8, CTX-M-9, CTX-M-25 and KLUC. CTX-M groups 1, 2, 8 and 9 are the four most common CTX-M groups harbored by Gram-negative pathogens [7]. Extraintestinal infections are usually opportunistic in nature and often occur due to conditions such as dysfunctional urinary tract or systemic immunocompromise [8]. *E. coli* isolates in general consist of a clonal population comprising of seven major phylogenetic groups (A, B1, B2, C, D, E and F). These phylogenetic groups have varying propensities to virulence factors, association with host and the environment [9]. Moreover, the presence of specific set of virulence factors is attributable to the ability of *E. coli* strains to cause disease by various pathotypes that are broadly classified into intestinal and extraintestinal pathogenic *E. coli* [9]. Characterization of clinical *E. coli* strains with respect to their pathotypes, AMR profiles and virulence gene will promote understanding of the epidemiology of such infections. 

AMR is a global problem that has real implications for human health [10]. Moreover, developing countries such as Bangladesh are highly vulnerable to the growing threat of AMR due to its high population density, inadequate sanitation and excessive use of antimicrobials in clinical, animal and agriculture sectors [11]. This together with the high burden of infectious diseases makes it pertinent to elucidate the molecular characteristics of circulating pathogens for which the data available are sparse. Therefore, this study aimed to assess the population structure, virulence potential and antimicrobial susceptibility profiles of clinical *E. coli* isolates from Dhaka, Bangladesh.

## 2. Results

### 2.1. Multidrug Resistance

Disc diffusion test entailing 16 antibiotics showed a high level of resistance among the *E. coli* isolates analyzed. The frequencies of resistance/susceptibilities are mentioned in Table 1. Overall, 69% (88/128) of isolates were resistant to at least three antibiotic classes, indicating they were multidrug-resistant. In fact, around 50% of isolates were resistant to at least seven antibiotics, with resistance ranging up to 15 antibiotics. Interestingly, the *E. coli* isolates assessed were highly susceptible to following antibiotics, carbapenems (meropenem (94%) and imipenem (91%)), amikacin (95%) and nitrofurantoin (89%). The *intI1* gene corresponding to class 1 integron was present in 27 (21%) of the 128 isolates analyzed. The IncF replicon types including FIA and FIB each were detected in over half (55%) of the *E. coli* isolates (Table 2).

### 2.2. Group B2 Was the Most Prevalent Phylogenetic Group

With respect to phylogenetic groups, most *E. coli* isolates belonged to the pathogenic phylogroup B2 (46%), followed by groups B1 (16%), D (10%), C (7%), F (9%), A (5%) and E (2%) (Table 2). Strains belonging to three phylogroups C, D and E strongly correlated with the MDR (78%, 85% and 100%, respectively) and ESBL production (89%, 69% and 100%, respectively). However, a majority of B2 phylogroup (68%) isolates were found to be MDR. All the three major ESBL genes detected [*bla_CTX-M_* (with extended hydrolyzing activity against extended-spectrum cephalosporins), *bla*_TEM_ (that has limited activity against newer cephalosporins) and *bla*_OXA-1_ (that has limited activity against newer cephalosporins)] were identified in multiple phylogenetic backgrounds.

### 2.3. Over Half of the Isolates Were ESBL Producers with bla_CTX-M-15_ Gene Being Dominant

Genotypically (taking into account the presence of, CTX-M-1, -2 and -9 groups, *bla*_CTX-M-15_, *bla*_TEM_ and *bla*_OXA-1_) the overall prevalence of ESBL *E. coli* isolates was 66% (84/128 isolates carried at least one ESBL gene screened), whereas phenotypically (using CHROMagarESBL) only 51% isolates (65/128) were found to be ESBL producers. The difference observed between results of genotypic and phenotypic ESBL detection might be due to lower sensitivity of phenotypic methods. The *bla*_CTX-M-15_ gene was significantly associated with the ESBL phenotype (*p* = 0.004), with 55 isolates carrying the *bla*_CTX-M-15_ gene out of 65 ESBL-producing *E. coli*. In addition, the most prevalent ESBL genotype was the CTX-M-1 group (52%), particularly the *bla*_CTX-M-15_ gene (Table 2). The other ESBL genotypes detected were *bla*_OXA-1_ (17%), *bla*_TEM_ (20%), CTX-M-9 group (3%) and CTX-M-2 group (1%). Multiple β-lactamase genes in combinations of *bla*_CTX-M-15_ or *bla*_OXA-1_ and *bla*_TEM_ were detected in 23% of the isolates. 

### 2.4. Molecular Characteristics of Carbapenem Resistant Strains

Carbapenemases are the β lactamase enzymes that can hydrolyze carbapenems including all other β lactam antibiotics. The most common carbapenem resistance genes are *bla*_NDM-1_, *bla*_OXA-48_, *bla*_KPC-2_, *bla*_VIM-1_ and *bla*_IMP_. Overall, 7% of *E. coli* isolates assessed in this study were carbapenem-resistant (nine out of 128 *E. coli* isolates carried at least one carbapenem resistance gene). The most prevalent carbapenem resistance gene was *bla*_NDM-1_ (5%, 6/128), followed by *bla*_OXA-48_ (2%, 2/128) and *bla*_IMP_ (1%, 1/128) (Table 2). Interestingly, in this study, the genes *bla*_VIM-1_ and *bla*_KPC-2_ were not identified. All NDM-1 positive *E. coli* were MDR; in fact, a majority of them (5/6) were pandrug resistant and co-harbored the *bla*_CTX-M-15_ gene. All nine carbapenem-resistant isolates were phenotypically resistant to an average of 11.6 antimicrobial agents and 4.2 different antibiotic classes (Table 3). Only 44% (4/9) isolates carrying carbapenemase genes showed resistance to meropenem and imipenem by disc diffusion methods. These strains mainly belonged to non-pathogenic phylogroups and none of them was affiliated to any of the four major STs screened. 

### 2.5. All Four Major Sequence Types (STs) Were Detected

Analysis of major *E. coli* sequence types revealed that ST131 was the most prevalent ST, accounting for 22% (28/128) of the studied isolates; 100% of the ST131 *E. coli* isolates were associated with the phylogroup B2 and fluoroquinolone (ciprofloxacin) resistance; and 82% (23/28) of ST131 *E. coli* harbored *bla*_CTX-M-15_ gene. The other prevalent STs were ST95 (9%), followed by ST69 (4%) and ST73 (3%) (Table 2). All (100%) of ST131 and ST95 isolates were identified as phylogroup B2 strains. All these four major STs were predominantly from ExPEC pathotype. 

### 2.6. Pathotype Assignment

Overall, 52% (66/128) of all *E. coli* isolates were potentially pathogenic, as defined by the presence of characteristic virulence markers (Table 2). ExPECs (isolates positive for three or more ExPEC virulence markers) constituted 41% (52/128) of all *E. coli* isolates. The prevalence of ExPEC in urine isolates was 44% (45/103), whereas the prevalence in isolates cultured from other body fluids was 28% (7/25). The diarrheagenic variants constituted 11% (14/128) of all *E. coli* isolates. Pathogenic *E. coli* isolates were significantly associated with the ESBL-genotype *bla*_CTX-M-15_ and phylogenetic group B2 (*p* < 0.05 for both). The proportion of MDR was higher in pathogenic *E. coli* variants compared to non-pathogenic *E. coli* isolates (*p* < 0.05). In addition, the *pks* pathogenicity island that encodes a genotoxin called colibactin was detected in 9% (11/128) of the *E. coli* isolates. The presence of *pks* pathogenicity island strongly correlated with ExPEC pathotype as all nine isolates were classified as ExPEC. These 11 strains majorly belonged to ST95 (5/11) and ST73 (3/11), and none were from ST131. 

## 3. Discussion

Extraintestinal pathogenic *E. coli* (ExPEC) is the most frequent cause of community-acquired UTIs accounting for significant morbidity, mortality and health care costs. In this study, we carried out a comprehensive molecular characterization of clinical *E. coli* isolates from a major referral diagnostic center (icddr,b) in Dhaka and demonstrated the presence of major ESBL-*E. coli* clones similar to those reported in different parts of the world [12]. Overall, high resistance rates to antibiotics including cephalosporins, fluoroquinolone and trimethoprim were found, as these are the most frequently used antibiotics to treat patients with UTIs, whereas the resistance rates to aminoglycosides were moderate and those to carbapenems and nitrofurantoin were low in the studied isolates. These observations were consistent with earlier studies from Bangladesh [13]. 

In our study, 41% isolates were classified as ExPEC and 11% were identified as diarrhoeagenic *E. coli* based on the presence of predictive virulence factors. The remaining 48% isolates could not be typed as ExPEC or diarrhoeagenic *E. coli*. These results are similar to those described by another study [14]. Further, a majority of these isolates belonged to phylogenetic groups B2, B1 and D. Phylogroups B2 and D are considered to be associated with more virulent strains frequently linked to infections [15]. However, the presence of B1 group as the second most predominant phylogroup in our study isolates suggest colonization of urinary tract with strains from gastrointestinal tract because B1 and A groups mostly belong to commensal *E. coli* [15]. Phylogroups B2, D, C and E were comprised in majority of MDR isolates rather than the commensal phylogroups A and B1, an observation in line with other studies [13,16]. The two most frequently identified *E. coli* lineages in our studied isolates were ST131 and ST95; other STs detected include ST73 and ST69. This is in line with previous reports that showed the predominance of sequence types, 131, 95, 73 and 69 among *E. coli* obtained from UTI and blood stream infections [17,18]. Similar to the previous report, our results show that ST95 isolates had low prevalence of antibiotic resistance [19]. The high prevalence of ST131 isolates in this study is not surprising, as ST131 has emerged as the most predominant high-risk clone among extraintestinal infections worldwide [20]. The presence of high MDR rates together with extensive virulence gene content in these ST131 *E. coli* isolates are considered a double threat. However, most *E. coli* isolates irrespective of phylogroups and pathotypes were susceptible to amikacin, meropenem and nitrofurantoin antibiotics. 

The majority of ESBL-producing *E. coli* isolates in this study harbored the *bla*_CTX-M-15_ gene similar to previous studies [21]. The increasing predominance of the *bla*_CTX-M-15_ allele together with the co-occurrence of other ESBL genes might probably offer the bacteria a selective advantage in overcoming multiple antibiotic pressure. Similarly, the majority of isolates from ST131 lineage identified in this study were CTX-M-15 producing. The majority of CTX-M-15 producing *E. coli* isolates were found to be MDR. In contrast, a majority of these isolates were susceptible to amikacin and nitrofurantoin antibiotics, suggesting that these antibiotics can still be a reliable option for empirical treatment of urinary tract infections. This is similar to data presented by other studies [22,23]. Carbapenems are antibiotics used to treat life-threatening infections caused by MDR Enterobacteriaceae such as *E. coli* [24]. The emergence of carbapenem-resistant bacterial isolates is of great medical concern globally. The majority of carbapenem-resistant isolates of this study carried the *bla*_NDM-1_ gene followed by *bla*_OXA-48_, similar to previous studies from Southeast Asia [24,25]. Most of the carbapenem resistant isolates in this study were resistant to a broad-spectrum of antibiotics (pan-drug resistant). None of the antibiotics out of the 16 antibiotics in our panel was 100% effective against these isolates. This is the reason why the standard treatment of carbapenem-resistant Enterobacteriaceae (CRE) include polymixin based combination therapy; other antibiotics include fosfomycin and tigecycline [26]. However, these are also less suitable for their use in neonates and infants [26]. In view of this, it is very important to strengthen and establish the epidemiological surveillance of CRE. 

The pathogenicity of extraintestinal pathogenic *E. coli* is based on the presence of several virulence factors that help bacteria to establish a successful infection, assimilate essential nutrients and disseminate in the urinary tract. The most frequently identified virulence factors in our isolates were associated with siderophores and protectins followed by adhesins. These results are consistent with results from previous studies which showed high frequency of siderophores, adhesins and capsular polysaccharides [4,27]. The isolates harboring ExPEC associated virulence factors were predominantly affiliated to B2 phylogroup and MDR phenotype. Another commonly found gene in our isolates was *hlyD* gene associated with toxins and is required for the dissemination of pathogens in the urinary tract. We also detected the presence of a genotoxin encoded by *pks* pathogenicity island in 9% (11/128) isolates. This is in agreement with the findings of others [28]. The presence of *pks* island was strongly correlated with ExPEC pathotype, besides these strains showed low rates of antibiotic resistance, this is similar to data presented in other reports [28]. 

In conclusion, this study provides evidence for prevalence and widespread dissemination of MDR mainly via phylogenetic group B2 strains and coexistence of multiple ESBL-genes (CTX-M-group1, *bla*_TEM_ and *bla*_OXA-1_) in clinical *E. coli* isolates. Notably, *E. coli* ST131, the pandemic ExPEC lineage, was identified in moderate proportion among ExPEC strains in this study. Our results reveal a moderate prevalence of carbapenamase gene carrying clinical *E. coli* isolates. Our results also suggest that several antibiotics such as meropenem, imipenem, amikacin and nitrofurantoin can be used to effectively treat infections in these settings. Further work is required at the genomics level to elucidate the molecular mechanisms shaping the evolution of clinical *E. coli* isolates with respect to acquisition of newer resistance and virulence determinants. Our data highlight the significance of longitudinal surveillance of pathogens and molecular epidemiology in different settings to understand the local epidemiological scenario and contemplate the global epidemiology on AMR. 

## 4. Methods

### 4.1. Sample Collection

In this retrospective study, 128 *E. coli* isolates were analyzed, of which 103 were cultured from urine specimen of suspected urinary tract infected patients and 25 isolates were cultured from other body fluids (Table S1). These isolates represented 1% of all the *E. coli* isolates cultured at the Clinical Microbiology and Immunology Laboratory of International Centre for Diarrhoeal Disease Research, Bangladesh (icddr,b) between March 2018 to July 2019. Identification of *E. coli* isolates was performed by routine biochemical tests that included; Kliger’s iron agar, motility indole urease, Simmons citrate agar, oxidase and ONPG tests. Bacterial isolates were preserved at −20 °C in trypticase soy broth containing 25% glycerol. 

### 4.2. Antimicrobial Susceptibility Testing and ESBL Detection

Antibiotic sensitivity testing was performed for all *E. coli* isolates by standard disc diffusion protocol using Mueller–Hinton agar as per the guidelines of Clinical Laboratory Standard Institute. The antimicrobial agents tested were ampicillin (10 µg), amoxiclav (3 µg), piperacillin–tazobactam (100/10 µg), gentamicin (10 µg), amikacin (30 µg), cotrimoxazole (25 µg), nalidixic acid (30 µg), ciprofloxacin (5 µg), nitrofurantoin (200 µg), cefuroxime (30 µg), ceftriaxone (30 µg), ceftazidime (30 µg), cefixime (5 µg), cefepime (30 µg), imipenem (10 µg) and meropenem (10 µg). *E. coli* ATCC 25922 strain was used for quality control. Screening of ESBL production was done using CHROMagarESBL^TM^ (Paris, France) [29].

### 4.3. Molecular Characterization of Antimicrobial Resistance

Molecular determination of antimicrobial resistance was performed by PCR based amplification of genes using gene specific primers described previously. Isolates were screened for ESBL genes (CTX-M-1, -2 and -9 groups, *bla*_CTX-M-15_, *bla*_TEM_ and *bla*_OXA-1_) [9], carbapenem resistance genes (*bla*_NDM-1_, *bla*_KPC-2_, *bla*_OXA-48_, *bla*_VIM-1_ and *bla*_IMP_), [30] class 1 integron gene (*intl1*) [4] and plasmid replicon types (FIA and FIB) [4].

### 4.4. Determination of Phylogroups and Major E. coli Sequence Types

All isolates were screened for phylogenetic groups A, B1, B2, C, D, E and F using one quadraplex PCR targeting *chuA*, *yjaA*, *TspE4.C2* and *arpA* and two singleplex PCRs targeting *arpAgpE* and *trpA* on DNA template obtained by boiling lysis method [31]. Strains were assigned to the above seven phylogenetic groups based on the criteria described by Clermont and colleagues [31]. The four major *E. coli* sequence types (STs), namely 69, 73, 95 and 131, were determined using a rapid PCR based multiplex assay described by Doumith et al. [17].

### 4.5. Detection of pks-Genomic Island

All isolates were screened for the presence of *pks* island by using PCR primers for two representative genes of the genomic island to amplify one flanking region (*clbB*) and one internal region (*clbA*) at annealing temperature of 65 and 45 °C, respectively, using standard PCR conditions [28]. 

### 4.6. Pathotype Assignment

For analysis of virulence factors that assign isolates to different intestinal pathotypes, PCRs targeting gene encoding: shigatoxin (*stx*) for enterohemorrhagic *E. coli* (EHEC), intimin (*eae*) for enteropathogenic *E. coli* (EPEC), invasion plasmid antigen (*ipaH*) for enteroinvasive *E. coli* (EIEC), heat stable (*elt*) and heat labile enterotoxin (*est*) for enterotoxigenic *E. coli* (ETEC), aggregative virulence regulator (*aggr*), L-aspartyl-tRNA (*aspU*) and plasmid segment of pCVD432 for enteroaggregative *E. coli* (EAEC) were used [32]. The following seven ExPEC associated virulence factors were screened: afimbrial adhesion (*afa*), F1C fimbriae (*focG*), iron acquisition system (*iutA*), cytolytic protein toxin (*hlyD*), P fimbriae (*papA*), group 2 polysaccharide capsule (*kpsMII*) and S fimbriae (*sfaS*) [9]. Strains having three or more of these genes were classified as ExPEC according to Johnson’s criteria [33]. 

### 4.7. Statistics

All statistical analyses were performed using SPSS. Fisher’s exact test was used to compare different proportions. *p*-values ≤ 0.05 were considered to be significant.

## Figures and Tables

**Table 1:** Antimicrobial resistance trends of clinical *E. coli* isolates to 17 antibiotics from March 2018 to July 2019.

Antibiotic Class	Antibiotics	No. of Strains Tested	No. of Resistant Strains (%)	No. of Sensitive Strains (%)
Aminopenicillin/β lactam	Ampicillin	128	107 (84)	21 (16)
β lactam/β lactamase inhibitor	Amoxiclav	128	70 (55)	58 (45)
	Piperacillin-Tazobactam	128	20 (16)	108 (84)
Aminoglycoside	Gentamicin	128	34 (27)	94 (73)
	Amikacin	128	7 (5)	121 (95)
Sulfonamide	Cotrimoxazole	128	76 (59)	52 (41)
Quinolone/Fluoroquinolone	Nalidixic Acid	125	104 (83)	21 (17)
	Ciprofloxacin	127	78 (61)	49 (39)
Nitrofuran	Nitrofurantoin	104	11 (11)	93 (89)
Cephalosporin	Cefuroxime	127	82 (65)	45 (35)
	Ceftriaxone	125	77 (62)	48 (38)
	Ceftazidime	128	66 (52)	62 (48)
	Cefixime	128	85 (66)	43 (34)
	Cefepime	127	59 (46)	68 (54)
Carbapenem	Imipenem	128	11 (9)	117 (91)
	Meropenem	126	8 (6)	118 (94)

**Table 2:** Molecular characteristics of 128 *E. coli* isolates analyzed including antimicrobial resistance, virulence and phylogenetic features.

Characteristics (Overall Positives n, %)		Gene	No. of Positives (%)
Antibiotic Resistance	ESBL (84, 66%)	*bla*_CTX-M-15_	66 (52)
		*bla*_CTX-M_ gp1	66 (52)
		*bla*_CTX-M_ gp2	1 (1)
		*bla*_CTX-M_ gp9	4 (3)
		*bla*_TEM_	26 (20)
		*bla*_OXA-1_	22 (17)
	Carbapenem (9, 7%)	*bla*_NDM-1_	6 (5)
		*bla*_IMP_	1 (1)
		*bla*_VIM-1_	0
		*bla*_KPC-2_	0
		*bla*_OXA-48_	2 (2)
Phylogenetic Groups		A	6 (5)
		B1	21 (16)
		B2	59 (46)
		C	9 (7)
		D	13 (10)
		E	2 (2)
		F	11 (9)
		Unknown group	7 (5)
Sequence types (48, 37%)		ST-69	5 (4)
		ST-73	4 (3)
		ST-95	11 (9)
		ST-131	28 (22)
Pathotypes	ExPEC (52, 41%)	*afa*	7 (5)
		*hlyD*	31 (24)
		*iutA*	79 (62)
		*focG*	4 (3)
		*kpsM II*	75 (59)
		*papA*	50 (39)
		*sfaS*	4 (3)
	EAEC (3, 2%)	*aggR*	1 (1)
		CVD432	3 (2)
		*aspU*	2 (2)
	ETEC (11, 9%)	*elt*	10 (8)
		*est*	2 (2)
	EIEC	*ipaH*	0
	EHEC	*stx*	0
	EPEC	*eae*	0
Class 1 integron (27, 21%)		*intl1*	27 (21)
Plasmid-replicon types (70, 55%)		FIA	70 (55)
		FIB	70 (55)
*pks* island (11, 9%)		*clbA*	11 (9)
		*clbQ*	11 (9)

**Table 3:** Antibiotic resistance profiles of *E. coli* isolates harboring carbapenem resistance genes.

Phylogroup	ESBL Genotype	CR ^b^ Genotype	AMR Profile ^c^	No. of Resistant Classes
B1	ND ^a^	*bla*_NDM-1_	AMP-COT-NIT-CIP-CXM	4
C	*bla*_CTX-M-15, TEM_	*bla*_NDM-1_	AMP-GEN-COT-NAL-NIT-CIP-CRO-CXM-AMX-AMC-TZP	5
F	*bla*_CTX-M-15, TEM_	*bla*_NDM-1_	AMP-GEN-COT-NAL-CIP-CRO-CAZ-IPM-CXM-AMX-CFM-AMC-MEM-FEP-TZP	5
D	*bla*_CTXM-15, TEM, OXA-1_	*bla*_NDM-1_	AMP-GEN-COT-NAL-CIP-CRO-CAZ-IPM-CXM-AMX-CFM-AMC-MEM-FEP-TZP	5
E	*bla*_CTXM-15, OXA-1_	*bla*_NDM-1_	AMP-NAL-NIT-IP-CRO-CAZ-CXM-CFM-AMC-FEP	3
D	*bla*_CTXM-15_	*bla*_NDM-1_	AMP-GEN-COT-NAL-NIT-CIP-CRO-CAZ-IPM-CXM-AMX-CFM-AMC-MEM-FEP-TZP	6
ND ^a^	*bla*_CTXM-15_	*bla*_IMP_	AMP-CRO-CXM-CFM-FEP	1
A	*bla*_CTXM-15, TEM, OXA-1_	*bla*_OXA-48_	AMP-COT-NAL-CIP-CRO-CAZ-CXM-AMX-CFM-AMC-FEP-TZP	3
B1	*bla*_CTXM-15_	*bla*_OXA-48_	AMP-GEN-COT-NAL-NIT-CIP-CRO-CAZ-IMP-CXM-AMK-CFM-AMC-MEM-FEP-TZP	6

^a^ ND, not detected; ^b^ Carbapenem resistance genotype; ^c^ AMP, ampicillin; GEN, gentamicin; COT, cotrimoxazole; NAl, nalidixic acid; NIT, nitrofurantoin; CIP, ciprofloxacin; CRO, ceftriaxone; CAZ, ceftazidime; IPM, imipenem; CXM, cefuroxime; AMK, amikacin; CFM, cefixime; AMC, amoxicillin-clavulanic acid; MEM, meropenem; FEP, cefepime.

## Supplementary Materials

The following are available online at https://www.mdpi.com/2079-6382/9/11/796/s1, Table S1: Demographic and molecular features of 128 *E. coli* isolates described in this study.
